# Wrong Tube: Tracheal Obstruction from Megaesophagus

**DOI:** 10.5811/cpcem.35603

**Published:** 2025-05-28

**Authors:** Adam Pearl, Abishek Roka

**Affiliations:** HCA Health Care Aventura Hospital, Aventura, Florida

**Keywords:** megaesophagus, respiratory distress, tracheal obstruction

## Abstract

**Case Presentation:**

Our patient presented in respiratory distress with stridor with the chief complaint of “inhaled a piece of pizza.” Foreign body airway obstruction algorithmic evaluation was followed but revealed megaesophagus compressing the trachea.

**Discussion:**

Megaesophagus is a disorder characterized by diffuse dilation with decreased peristalsis. It is commonly divided into congenital and acquired etiologies. Most documented cases have been secondary to longstanding achalasia and typically present as heartburn and regurgitation. It is imperative to keep broad differentials and avoid anchoring bias in patient evaluation. This rare case of respiratory distress secondary to a gastrointestinal issue highlights the importance of a broad differential and offers insight into a seldom reported occurrence.

## CASE PRESENTATION

A 33-year-old male arrived to the emergency department (ED) via emergency medical services (EMS) due to possible food inhalation. The EMS responders met the patient in the parking lot of a restaurant where his friends stated he began to choke while eating pizza. After noting apparent respiratory distress with audible stridor, EMS brought him to the ED. Upon presentation he was choking, had stridulous breath sounds, and was attempting to gag himself. Despite gross stridor, bilateral breath sounds were appreciated, and he was maintaining oxygen saturation of 96% on room air. There was no visible food in the oropharynx. The rest of his exam was unremarkable.

The patient’s voice was diminished to a whisper, but he was able to respond with basic answers as well as nod appropriately to questions. He confirmed that he had choked on pizza, denied any prior medical conditions, and denied any nausea or abdominal pain.

**Image 1:**
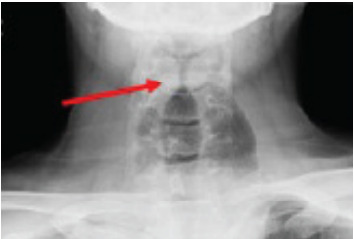
Anterior/posterior soft-tissue neck radiograph demonstrating proximal narrowing (arrow). Air seen in tissue surrounding trachea, originally thought to be pneumomediastinum, clinically correlates to esophagus.

Radiographs of the neck and chest were ordered to evaluate for possible food product in the respiratory tree. However, before radiographs were obtained, the patient gagged himself, had small emesis, and apparently cleared the possible obstruction. He began speaking in full sentences without stridor, the tachycardia resolved, and oxygen saturation improved to 100%. Soft-tissue neck radiograph demonstrated narrowing of the proximal trachea but did not show any signs of foreign body obstruction. In the setting of resolved symptoms, narrowing was originally thought to be possible laryngospasm (Image 1*)*. A thin radiolucency surrounding the cardiac silhouette raised concern for pneumomediastinum.

**Image 2:**
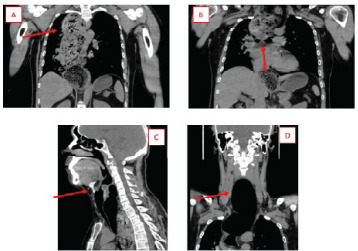
*A:* Coronal computed tomography (CT) chest demonstrating megaesophagus with impacted food products. *B:* Coronal CT chest demonstrating compressed carina with deviation of mid-trachea to the left. *C:* Sagittal CT neck demonstrating arrowing of trachea and anterior displacement of epiglottis. *D:* Coronal CT neck demonstrating dilated, air-filled esophagus.

Computed tomography (CT) was ordered to ensure the food product had been dislodged rather than advanced further in the bronchial tree, thus allowing air passage through the larger bronchioles. The CT was performed, and while imaging was uploading to the hospitals network the patient began tripoding as well as having significant stridor. Discussions about intubation ensued with further bronchoscopy from critical care physicians to remove food products. The CT images, uploaded while preparing for endotracheal intubation, demonstrated a significantly dilated esophagus with a large amount of ingested food material compressing the trachea proximally and displacing it distally (Image 2). No perforation was noted.

The decision was made to intubate for airway protection. The difficult-airway cart was at bedside given the degree of tracheal compression, its tortuous nature secondary to displacement, and overall high risk of the patient’s condition. A 7.5-millimeter endotracheal tube was placed, and propofol and fentanyl were used for sedation post-intubation. The patient remained hemodynamically stable, although clinical features of superior vena cava syndrome appeared. These symptoms resolved after nasogastric tube insertion with suctioning of gastric contents.

*CPC-EM Capsule*

What do we already know about this clinical entity?

*Achalasia is a relatively common upper gastrointestinal disorder. However, acute complication resulting in megaesophagus with tracheal obstruction is seldom*.

What is the major impact of the image(s)?

*These images demonstrate the gross anatomic pathology seen with megaesophagus and tracheal compression. The images provide an easy to comprehend schematic as well as a visual as to why our treatment was successful*.

How might this improve emergency medicine practice?

*This will improve emergency medicine by providing a differential, though uncommon, for acute respiratory distress. As emergency physicians, it is imperitive to avoid anchoring bias and keep a broad differential*.

The patient was admitted to the intensive care unit for further management. However, after initial stabilization, the decision was made to transfer him to a center where cardiothoracic surgery could be available during endoscopy.

### The Follow-up

**Image 3:**
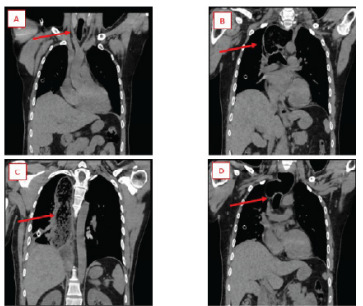
*A:* Coronal computed tomography (CT) chest demonstrating patent proximal trachea. *B:* Coronal CT chest demonstrating patent carina with no visible compression. *C:* Coronal CT chest demonstrating continued esophageal dilation with food content. *D:* Coronal CT chest demonstrating dilated esophagus overlying a patent, non-deviated trachea.

The patient was transferred and subsequently underwent endoscopy with decompression and removal of food burden. There were black patchy areas in the esophagus concerning for necrosis, and cardiothoracic surgery recommended total esophagectomy in the near future. He returned to baseline mental status, was extubated, and passed swallow evaluations. He was then returned to our facility for the remainder of his care. Repeat CT demonstrated significant alleviation of tracheal compression and deviation; however, esophageal distension was still prominent (Image 3). Repeat CT confirmed the likelihood of future esophagectomy.

## DISCUSSION

Megaesophagus is a disorder characterized by diffuse dilation with decreased peristalsis.^1^ It is commonly divided into congenital and acquired etiologies.^1^ Most documented cases have been secondary to longstanding achalasia and typically present as heartburn and regurgitation.^2^ Rarer etiologies have also been reported, such as secondary to Wilkie syndrome, although with similar presentation.^3^ While treatment and full evaluation will not occur in the ED, it is essential to recognize this rare entity early and intervene to protect the airway as the patient is at high risk for compromise.

In this case, even though the neck radiograph was ordered to evaluate for aspiration, the air surrounding the trachea was an early indicator that the esophagus may have been dilated and obstructed. Such a situation should prompt physicians to order further imaging. In the ED setting, megaesophagus would make few physicians’ list of differential diagnoses for stridor; however, in the mental algorithm, external compression of the trachea is a feasible cause. This serves as a reminder for emergency physicians to avoid anchoring bias, as an unlikely differential is bound to be the diagnosis from time to time.
